# Supplementary material for: A ferroptosis-targeting ceria anchored halloysite as orally drug delivery system for radiation colitis therapy
Source: Nat Commun. 2023 Aug 22;14:5083. doi: 10.1038/s41467-023-40794-w (PMC10444825; doi:10.1038/s41467-023-40794-w)
Supplement: Supplementary file 1 — Supplementary Information [file 41467_2023_40794_MOESM1_ESM.pdf]

Supplementary Information for

A Ferroptosis-targeting Ceria Anchored Halloysite as Orally Drug Delivery System for Radiation Colitis Therapy

Yue Feng,[1] Xiang Luo,[2,3,4] Zichun Li,[2,3,4] Xinjuan Fan,[5,6] Yiting Wang,[5,6] Rong-Rong He,[2,3,4]* Mingxian Liu[1]*

[1] Department of Materials Science and Engineering, College of Chemistry and Materials Science, Jinan University, Guangzhou 511443, China

[2] Guangdong Engineering Research Center of Chinese Medicine & Disease Susceptibility, Jinan University, Guangzhou 510632, China

[3] International Cooperative Laboratory of Traditional Chinese Medicine Modernization and Innovative Drug Development of Chinese Ministry of Education (MOE), College of Pharmacy, Jinan University, Guangzhou 510632, China

[4] Guangdong Province Key Laboratory of Pharmacodynamic Constituents of TCM and New Drugs Research, College of Pharmacy, Jinan University, Guangzhou 510632, China

[5] Department of Pathology, The Sixth Affiliated Hospital, Sun Yat-sen University, Guangzhou, 510655, China

[6] Guangdong Provincial Key Laboratory of Colorectal and Pelvic Floor Diseases, The Sixth Affiliated Hospital, Sun Yat-sen University, Guangzhou, 510655, China

*Corresponding author: rongronghe@jnu.edu.cn (Rong-Rong He), liumx@jnu.edu.cn (Mingxian Liu)

These authors contributed equally to this work: Yue Feng, Xiang Luo

These authors jointly supervised this work: Rong-Rong He, Mingxian Liu

Supplementary Figures

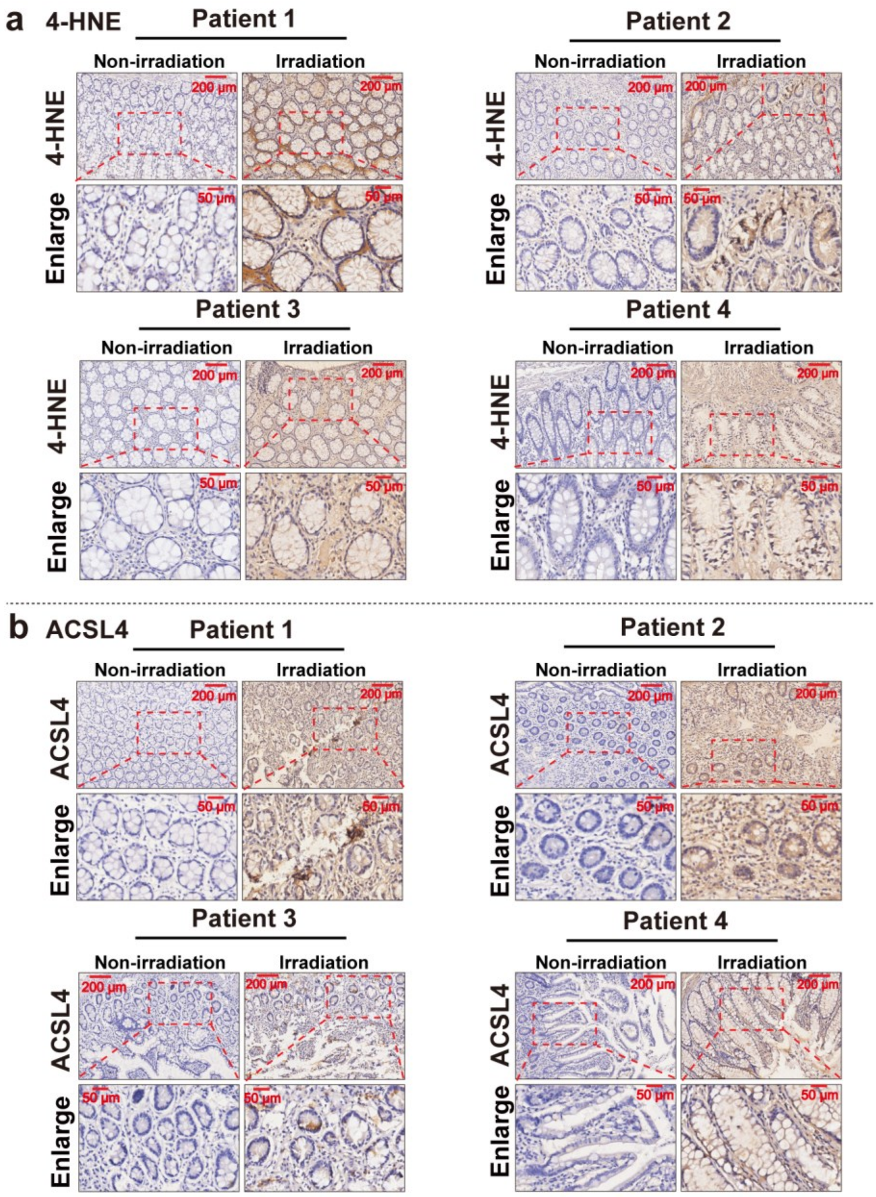

Supplementary Fig. 1. 4-HNE and ACSL4 are upregulated in human colon tissue from patients with radiation colitis. a, b) Representative immunohistochemical staining of 4-HNE and ACSL4 in colonic tissue samples from patients with radiation colitis. Scale bars: 200 μm and 50 μm. Immunohistochemistry results from five different fields of view showed similar trend. Experiments were repeated three times independently with similar results.

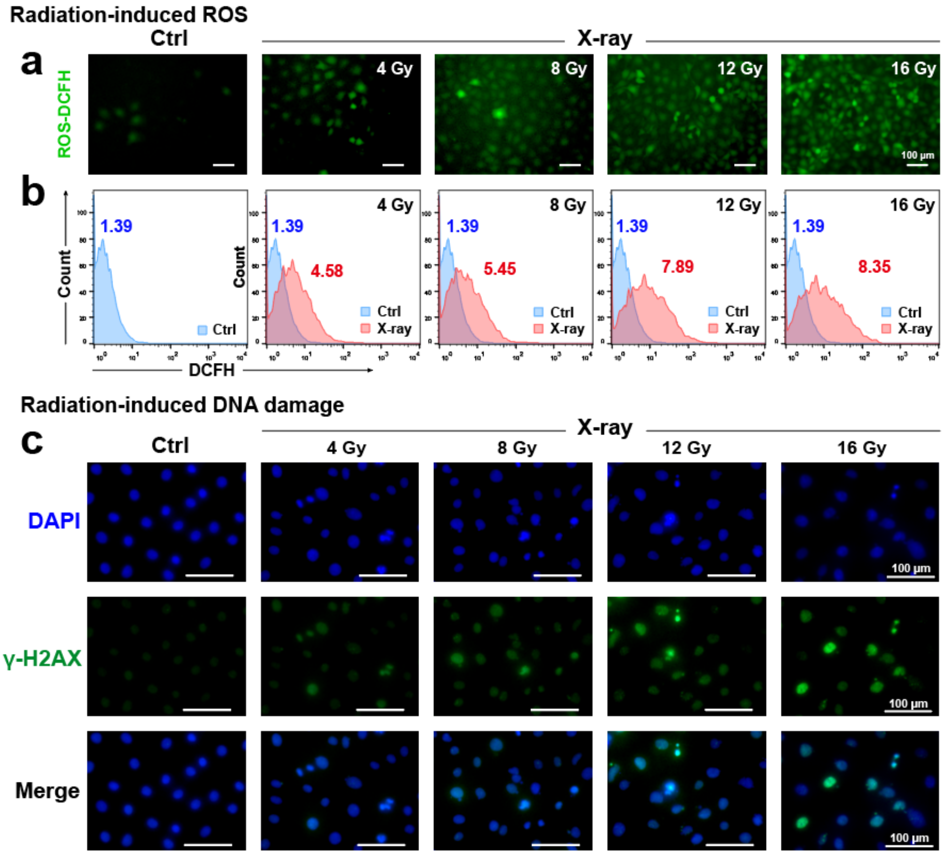

Supplementary Fig. 2. ROS production evaluated by DCF fluorescence (10,000 cells per tube were collected). **a**) Fluorescence microscopic images and **b**) representative flow cytometry plots of intracellular ROS in irradiated IEC-6 cells. **c**) Fluorescence microscopic images of irradiation-induced DNA double-strand breaks. Scale bars: 100 μm. Experiments were repeated three times independently with similar results.

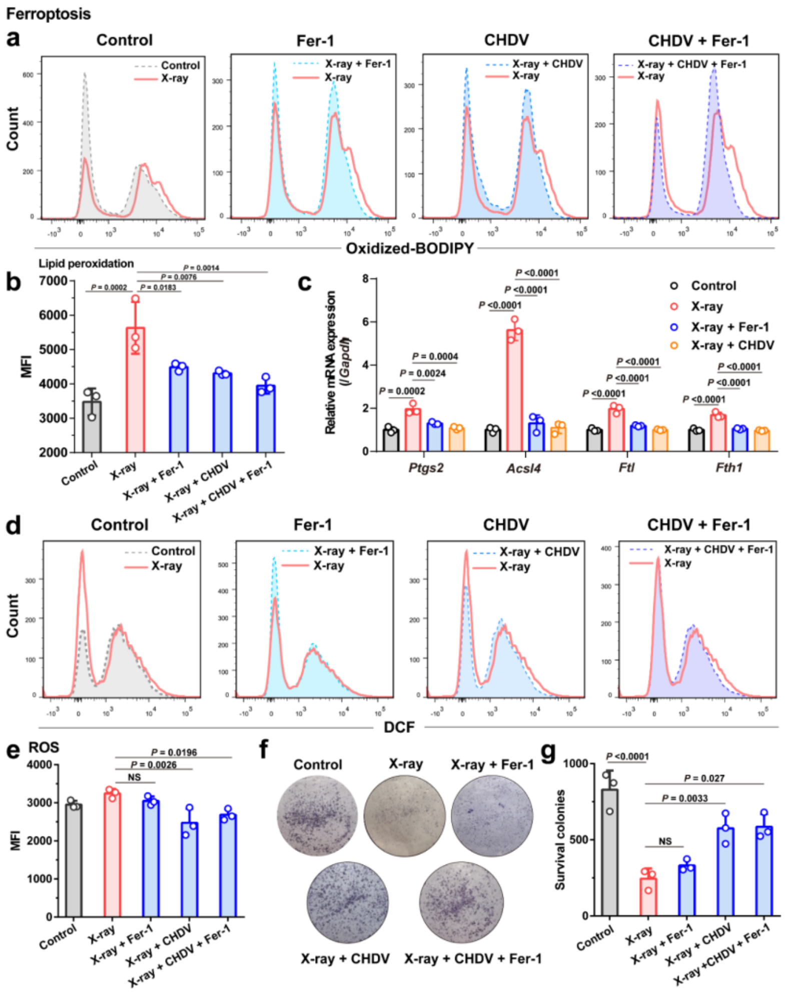

Supplementary Fig. 3. a) Lipid peroxidation assessment in IEC-6 cells pretreated with DMSO as normal control, 2 µM Fer-1, or 32 µg mL^{-1} CHDV for 24 h followed by exposure to 8 Gy of irradiation (10,000 cells per tube were collected). **b**) Histogram showing relative levels of lipid peroxidation by C11-BODIPY staining in the indicated cells, n = 3. **c**) Q-PCR analysis of ferroptosis-related genes (*Ptgs2*, *Acsl4*, *Ftl*, and *Fth1*) expression in IEC-6 cells pretreated with DMSO (control), 2 µM Fer-1, or 32 µg mL^{-1} CHDV for 24 h followed by exposure to 8 Gy of irradiation, n = 3. **d**) Intracellular total ROS indicated by DCFH-DA of IEC-6 cells pretreated with DMSO as normal control, 2 µM Fer-1, or 32 µg mL^{-1} CHDV for 24 h followed by exposure to 8 Gy of irradiation (10,000 cells per tube were collected), and **e**) the statistics of mean fluorescence intensity of DCF, n = 3. **f**) Crystal violet staining and **g**) quantification of the surviving colonies of IEC-6 cells irradiated by 8 Gy X-ray in different treatment groups, n = 3. Error bars are presented as mean ± SD. The data were analyzed by one-way ANOVA with Tukey's post hoc test. The experiments for **a**, **d**, and **f** were repeated three times independently with similar results. Source data are provided as a Source Data file.

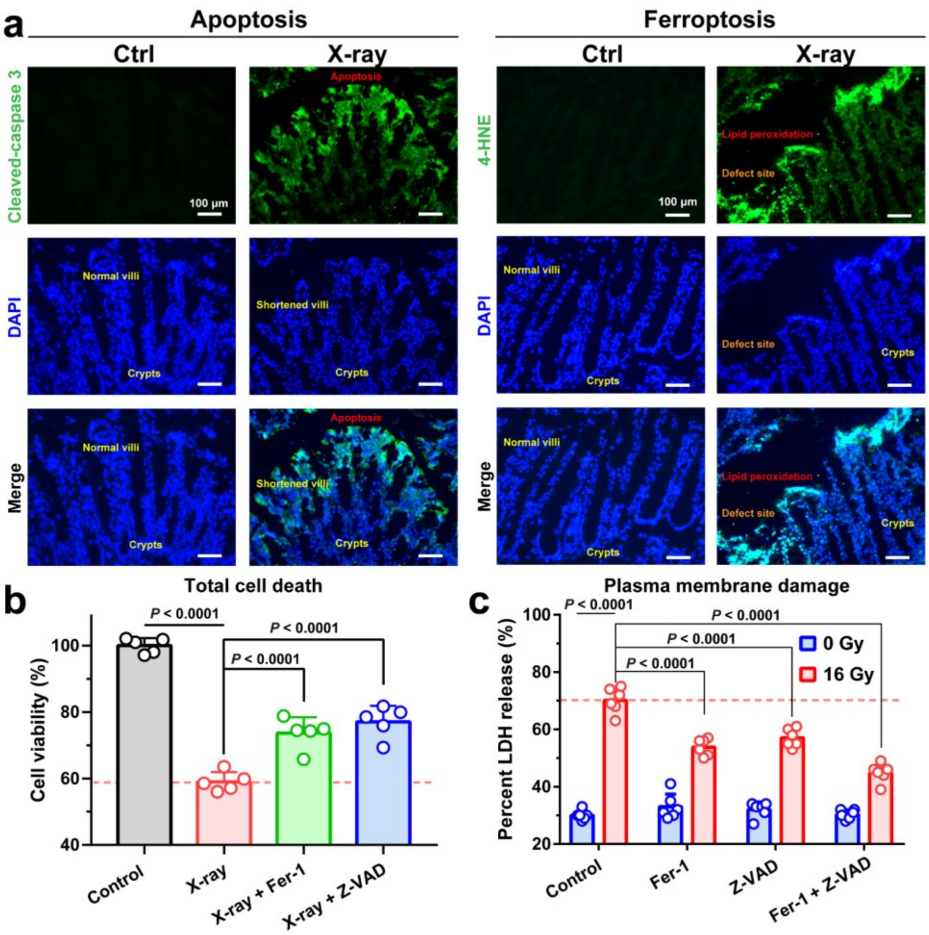

Supplementary Fig. 4. *In vivo* and *in vitro* tests for comparative studies of apoptosis/ferroptosis after exposure to irradiation. **a**) Immunofluorescence staining for apoptosis (Cleaved-caspase 3) and lipid peroxidation (4-HNE) in colon tissues. Cell nuclei were stained with DAPI to reveal villi and intestinal integrity. Scale bars: 100 μm. Experiment was repeated three times independently with similar results. **b**) Cell viability (n = 5) and **c**) LDH release (n = 5) of IEC-6 cells pretreated with DMSO as normal control, 2 μM Fer-1, 25 μM Z-VAD, or combination at 48 h after exposure to irradiation (16 Gy). The data are shown as mean ± SD. The data were analyzed by one-way ANOVA with Tukey's post hoc test. Source data are provided as a Source Data file.

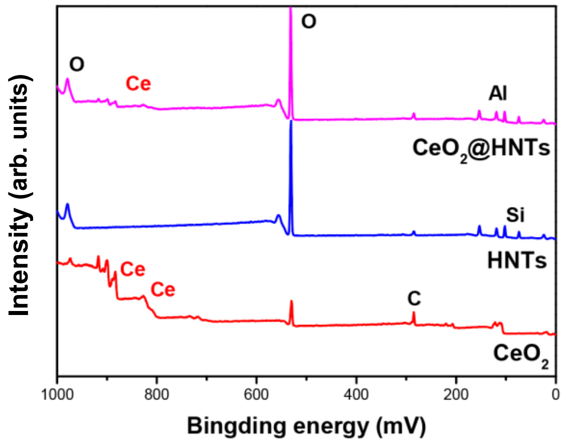

Supplementary Fig. 5. Survey XPS spectrum of CeO_2, HNTs, and CeO_2@HNTs. Source data are provided as a Source Data file.

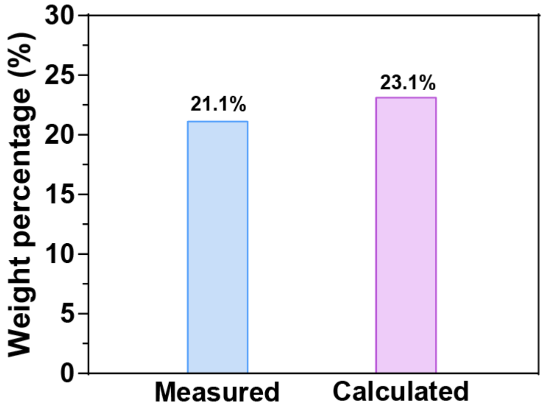

Supplementary Fig. 6. Loading (in weight percentage) of Ce on CeO_2@HNTs quantified by ICP-OES. Source data are provided as a Source Data file.

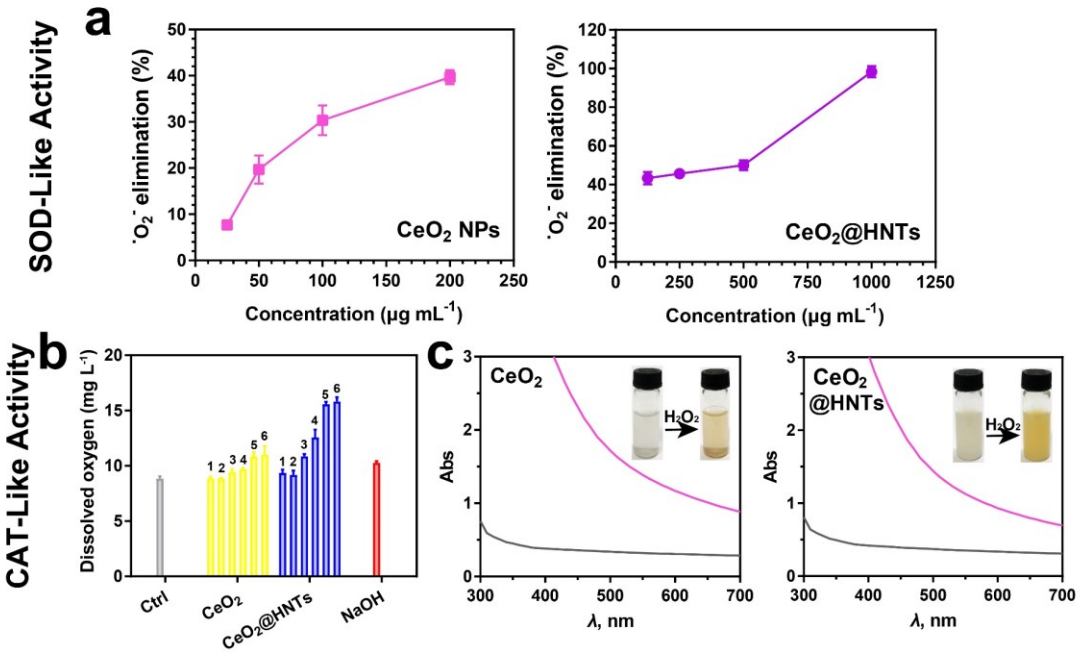

Supplementary Fig. 7. a) Percentage of superoxide radical elimination catalyzed by SOD-like activity of materials with increasing concentrations, n = 3. **b**) Oxygen generation from H_2O_2 catalyzed by CAT-like activity of materials with increasing concentrations, n = 3. Numbers 1 to 6 represent Ce concentrations of 25, 50, 100, 200, 400, and 800 μg mL^{-1}, respectively. **c**) UV-visible studies of CeO_2 and CeO_2@HNTs treated with H_2O_2. The data are shown as mean ± SD. Source data are provided as a Source Data file.

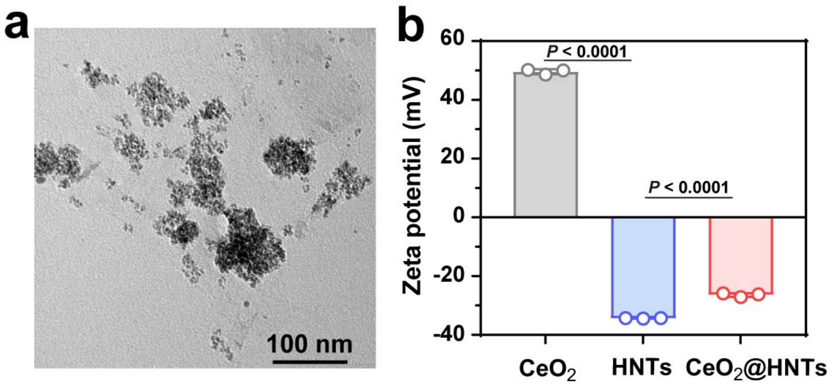

Supplementary Fig. 8. a) TEM image of CeO_2 agglomerates in aqueous media, scale bars: 100 nm. The experiment was repeated three times independently with similar results. **b**) Zeta potentials of CeO_2, HNTs, and CeO_2@HNTs ($n = 3$). The data are shown as mean ± SD. The data were analyzed by one-way ANOVA with Tukey's post hoc test. Source data are provided as a Source Data file.

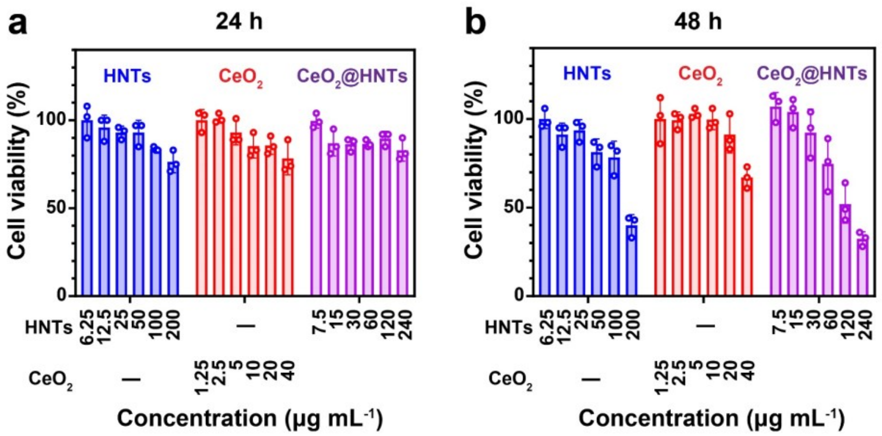

Supplementary Fig. 9. (**a**, **b**) Cell viability of IEC-6 cells after HNTs, CeO_2, and CeO_2@HNTs treatment for 24 hours or 48 h. The data are shown as mean ± SD (n = 3). Source data are provided as a Source Data file.

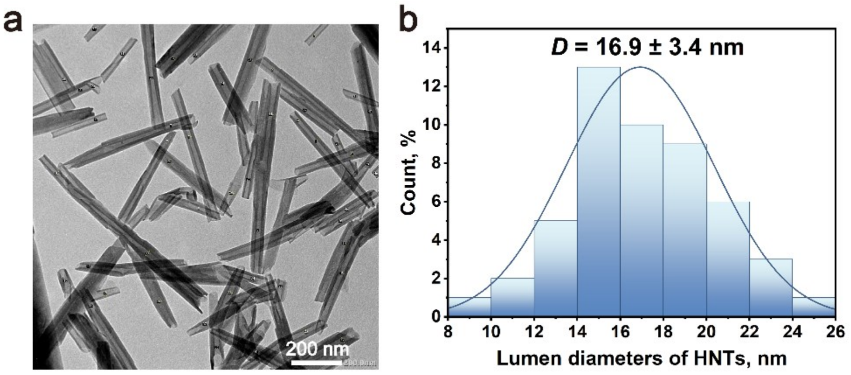

Supplementary Fig. 10. a) TEM image of HNTs, scale bars: 200 nm. The experiment was repeated three times independently with similar results. **b**) Their lumen diameters distribution. The data are shown as mean ± SD, n = 50. Source data are provided as a Source Data file.

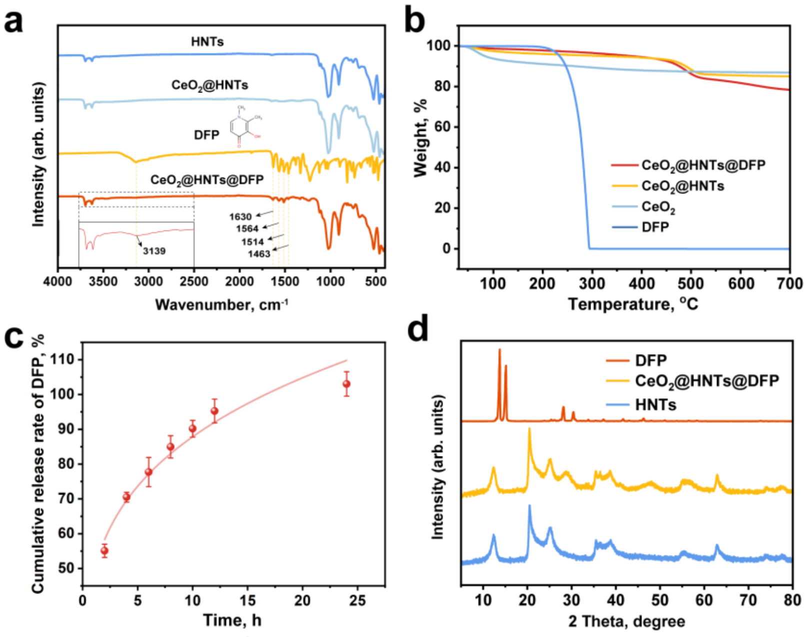

Supplementary Fig. 11. **a**) FTIR spectra and **b**) TGA curves of HNTs, DFP, CeO_2@HNTs, and CeO_2@HNTs@DFP. **c**) Korsmeyer-Peppas model fitting curve of DFP release at different time points, n =3. **d**) XRD patterns of HNTs, DFP, and CeO_2@HNTs@DFP. Error bars are shown as means ± SD. Source data are provided as a Source Data file.

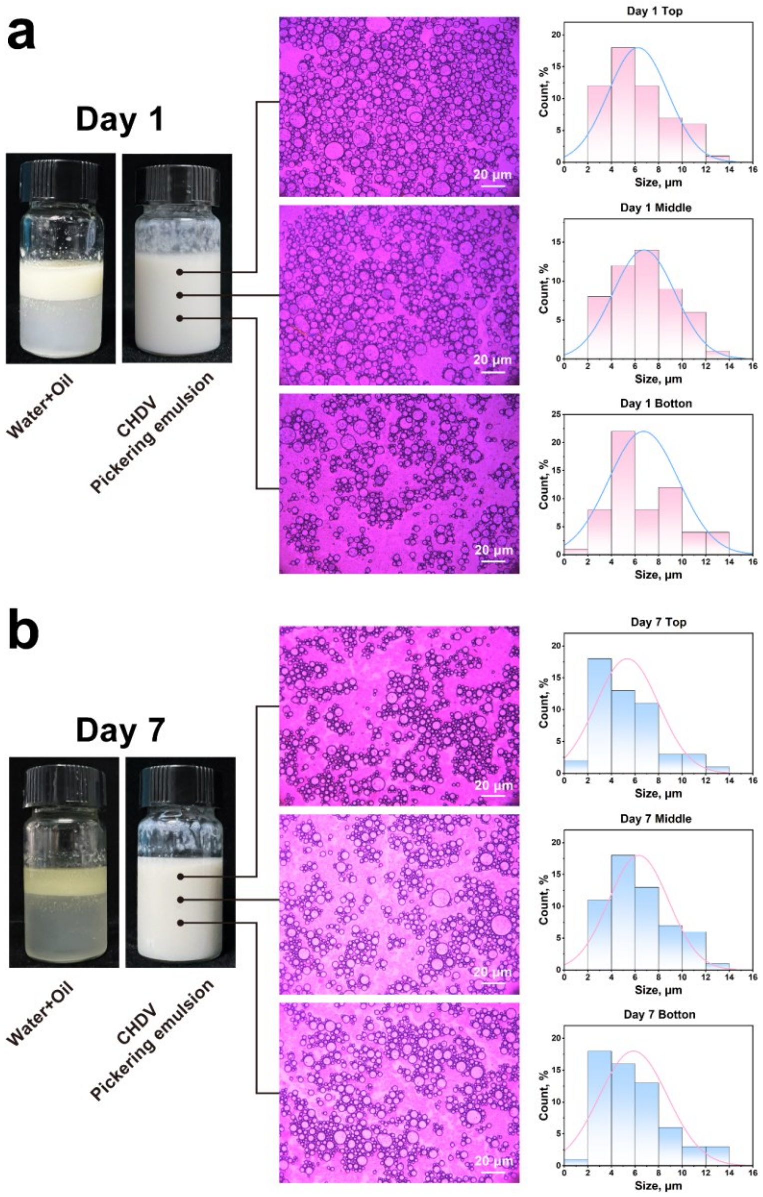

Supplementary Fig. 12. Polarizing microscope images and the size distribution charts of CHDV Pickering emulsion on **a**) day 1 and **b**) day 7. Scale bars: 20 µm. The data are shown as mean ± SD, n = 50. Source data are provided as a Source Data file.

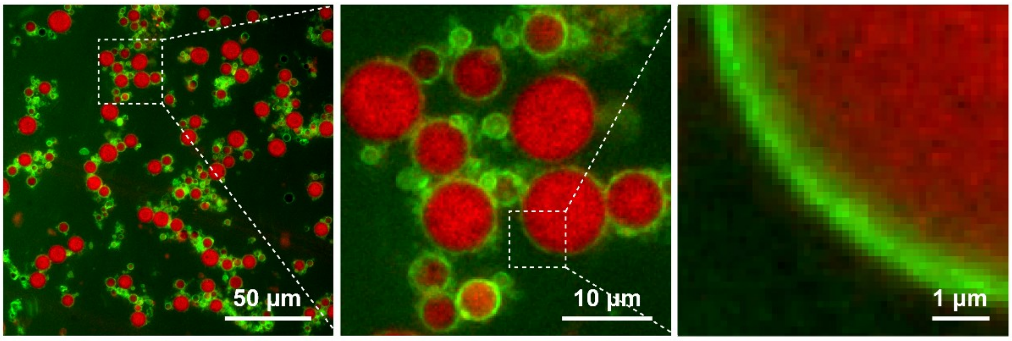

Supplementary Fig. 13. Fluorescence microscope images confocal microscopy images of "HNTs-stabilized" CHDV Pickering emulsions. FITC-labeled HNTs (green) can be observed in the outer ring of Nile Red-stained emulsion droplets (red). Experiment was repeated three times independently with similar results.

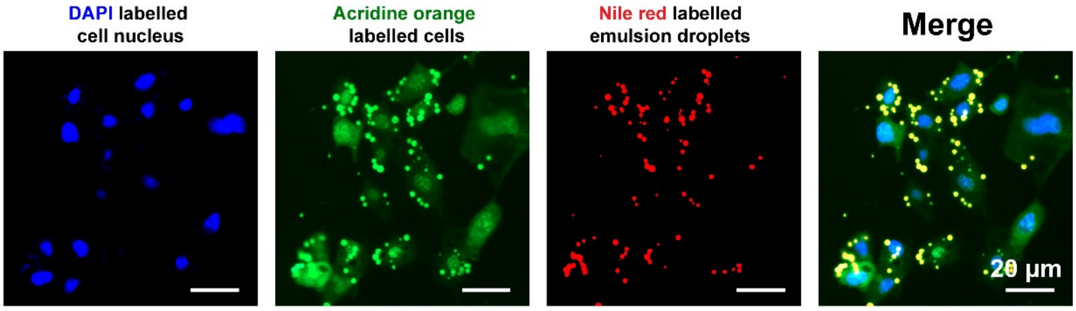

Supplementary Fig. 14. Fluorescence microscope images of emulsion droplets adhere to intestinal epithelial cells. Scale bars: 20 μm. Experiment was repeated three times independently with similar results.

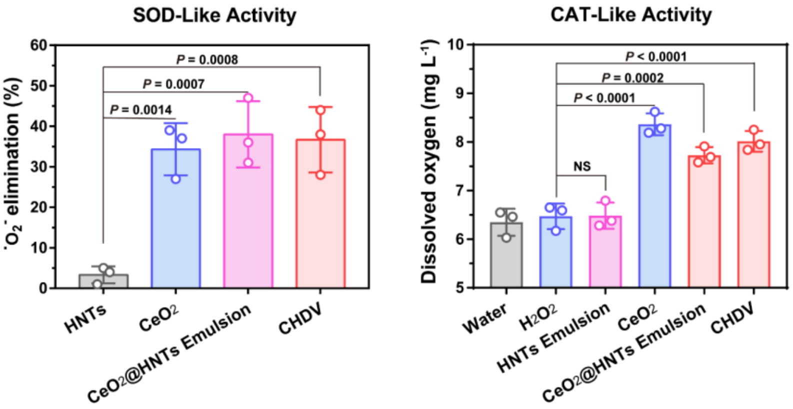

Supplementary Fig. 15. SOD-like and CAT-like activity of CHDV Pickering emulsion. Error bars are means ± SD, n = 3 independent repeats. The data were analyzed by one-way ANOVA with Tukey's post hoc test. Source data are provided as a Source Data file.

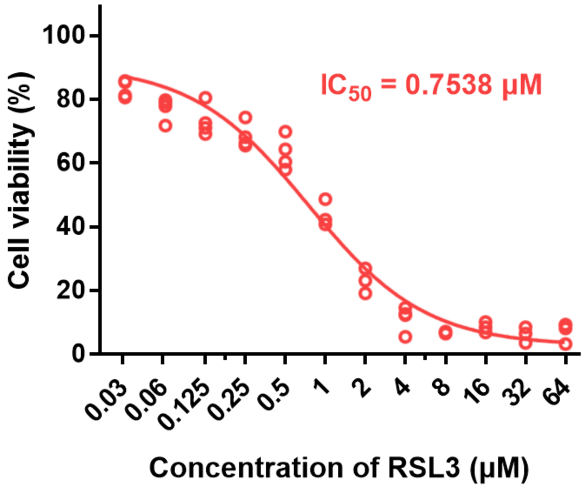

Supplementary Fig. 16. Cell viability and corresponding IC_{50} value of ferroptosis inducer RSL3 on IEC-6 cells after incubation for 6 hours. The data are shown as mean ± SD, $n = 4$. Source data are provided as a Source Data file.

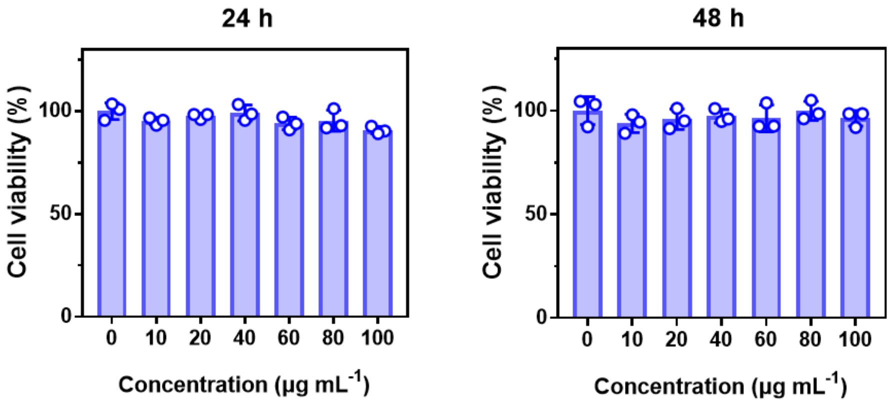

Supplementary Fig. 17. Cell viability after CHDV treatment without irradiation was tested by MTT assay. The data are shown as mean ± SD, $n = 3$. Source data are provided as a Source Data file.

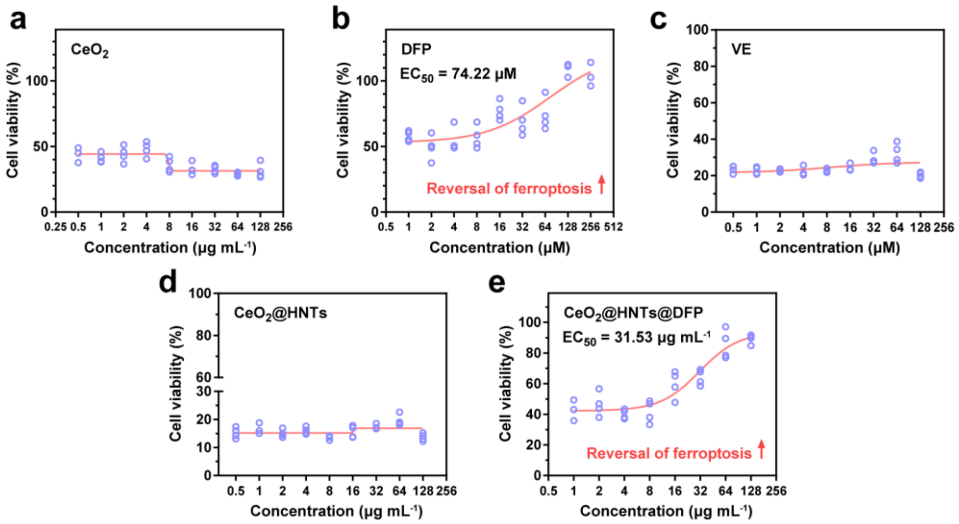

Supplementary Fig. 18. Cell viability showed that different components in CHDV including **a**) CeO_2, **b**) DFP, **c**) VE, **d**) CeO_2@HNTs, and **e**) CeO_2@HNTs@DFP reversed RSL3(0.8 μM)-induced ferroptosis. The data are shown as mean ± SD, $n = 4$. Source data are provided as a Source Data file.

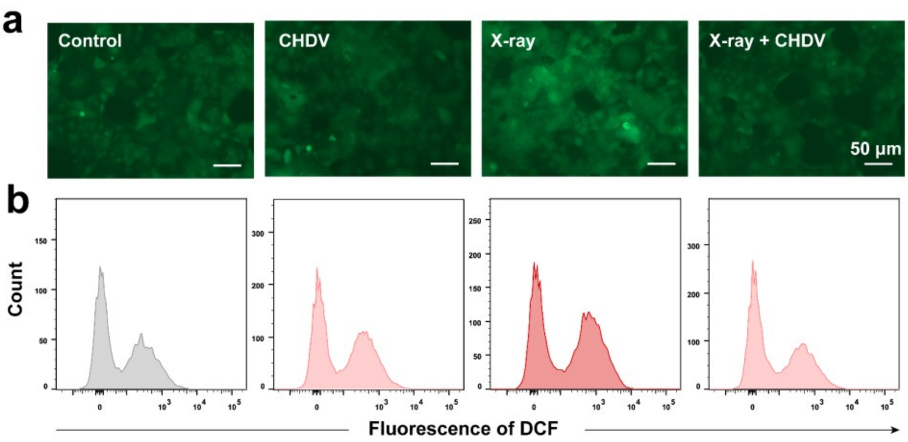

Supplementary Fig. 19. a) Fluorescence microscopic images (Scale bars: 50 μm) and **b**) representative flow cytometry histograms of intracellular ROS evaluated by DCF fluorescence with or without irradiation after CHDV treatment (10,000 cells per tube were collected). Experiments were repeated three times independently with similar results.

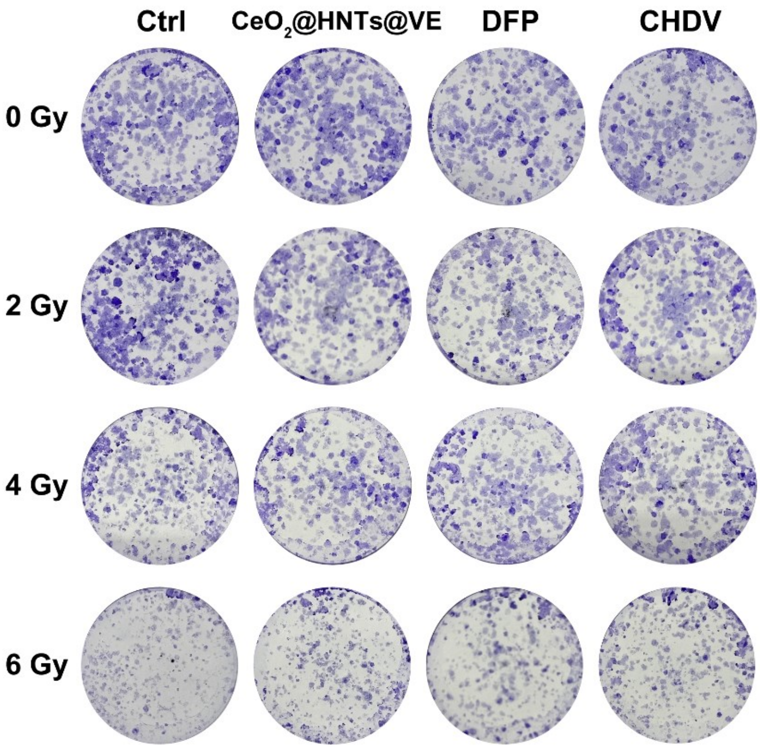

Supplementary Fig. 20. Representative clonogenic plates of IEC-6 cells expose to 0, 2, 4, or 6 Gy irradiation after treating with different components in CHDV. Experiment was repeated three times independently with similar results.

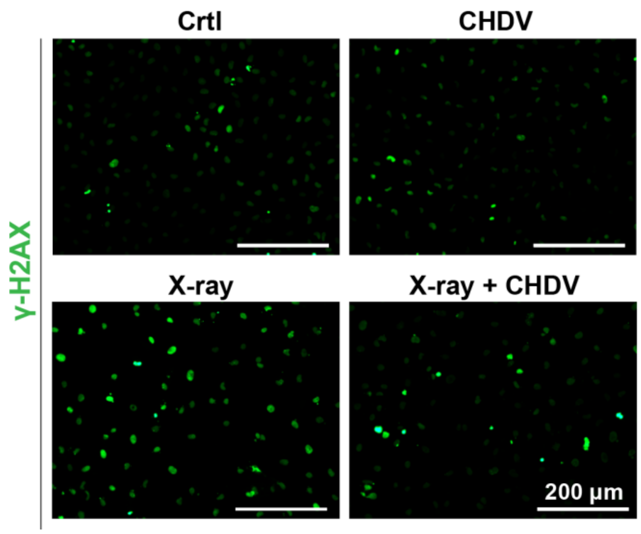

Supplementary Fig. 21. Fluorescence microscopic images of DNA double-strand breaks (evaluated via γ-H2AX) of IEC-6 cells after CHDV treatment with or without irradiation. Scale bars: 200 μm. Experiment was repeated three times independently with similar results.

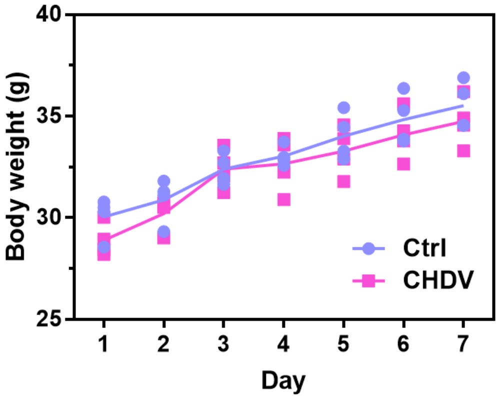

Supplementary Fig. 22. The body weight changes of mice experiencing 7 days of orally administered CHDV, with the same volume of saline as control ($n = 4$). Source data are provided as a Source Data file.

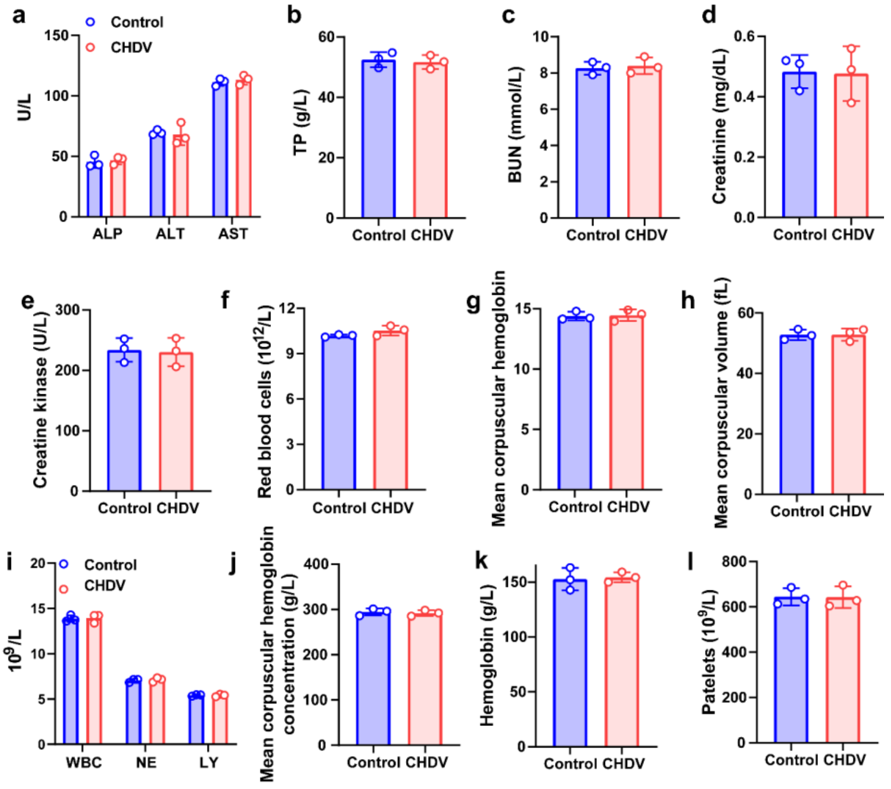

Supplementary Fig. 23. a-e) Blood biochemical and **f-l**) blood routine tests in mice after 7 days of CHDV administration. Blood biochemical indicators include: alkaline phosphatase (ALP), alanine aminotransferase (ALT), aspartate aminotransferase (AST), total protein (TP), blood urea nitrogen (BUN), creatinine, and creatinine kinase. Blood routine indicators include: red blood cell count, hemoglobin, mean corpuscular volume, white blood cell count (WBC), neutrophil count (NE), lymphocyte count (LY), platelets count, etc. The data are shown as mean ± SD, n = 3. Source data are provided as a Source Data file.

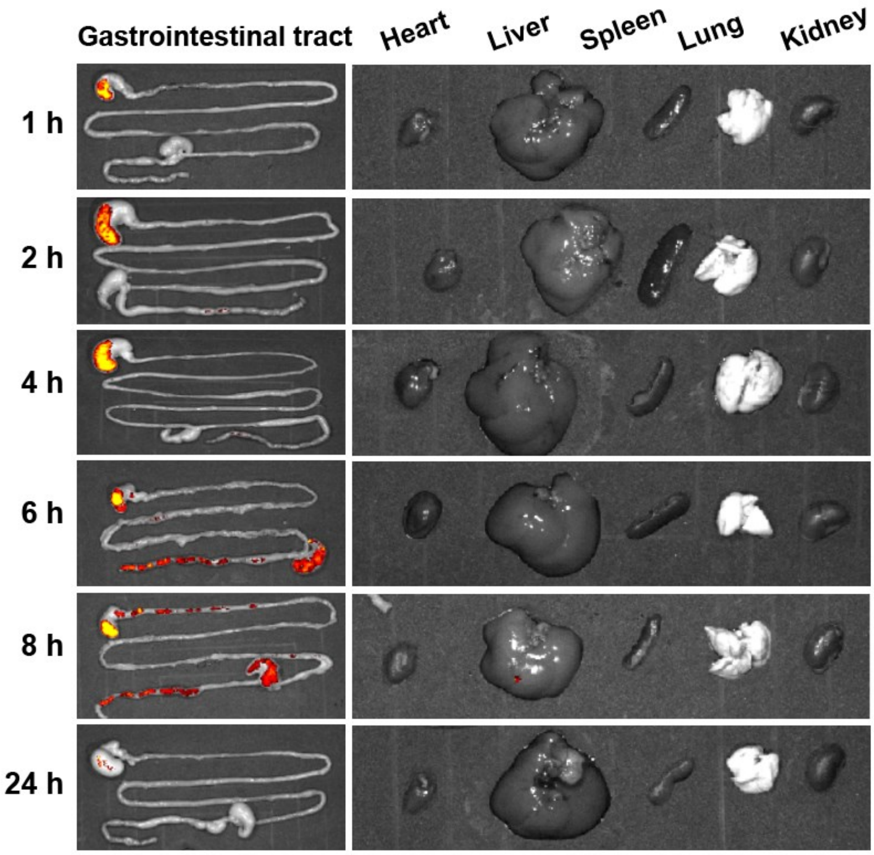

Supplementary Fig. 24. Biodistribution of CHDV (gastrointestinal tract and other organs) 1-24 hours after gavage. Experiment was repeated three times independently with similar results.

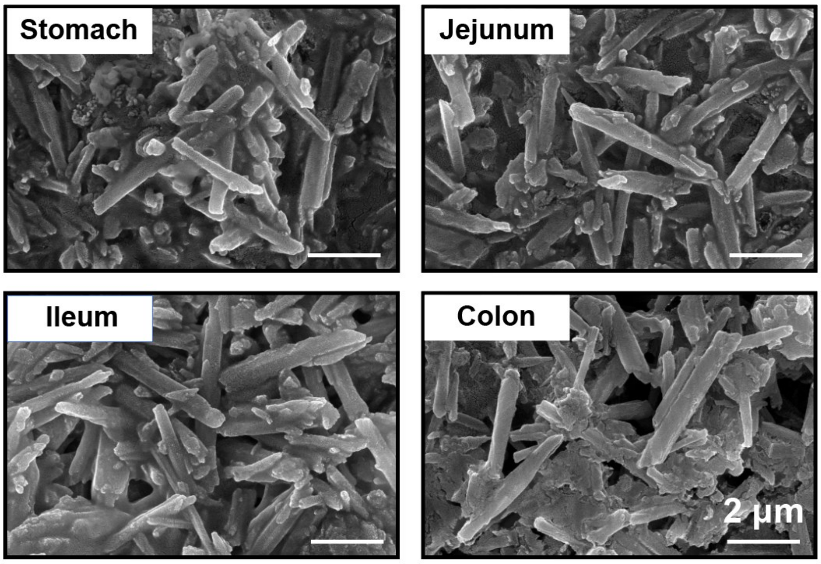

Supplementary Fig. 25. SEM images of intestinal contents obtained in different intestinal segments reveal the tubular structural integrity of HNTs. Scale bars: 2 μm. Experiment was repeated three times independently with similar results.

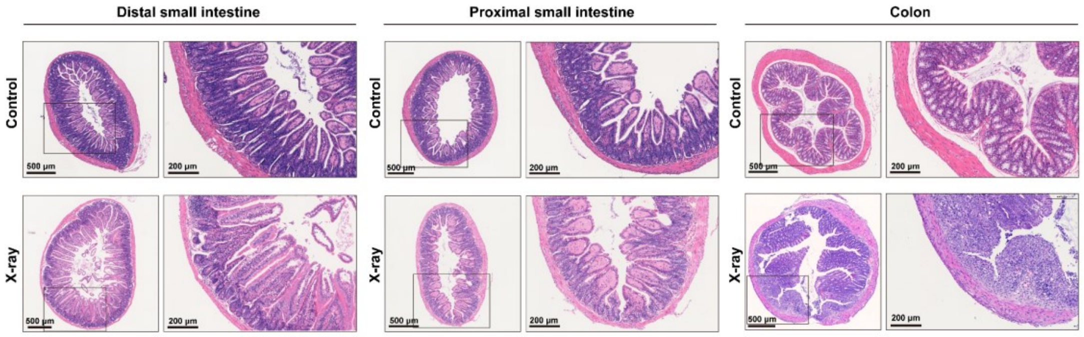

Supplementary Fig. 26. H&E staining of tissue sections of distal small intestine, proximal small intestine, and colon 7 days after irradiation exposure ($n = 3$ biologically independent animals). Scale bars: 500 µm and 200 µm. Experiment was repeated three times independently with similar results.

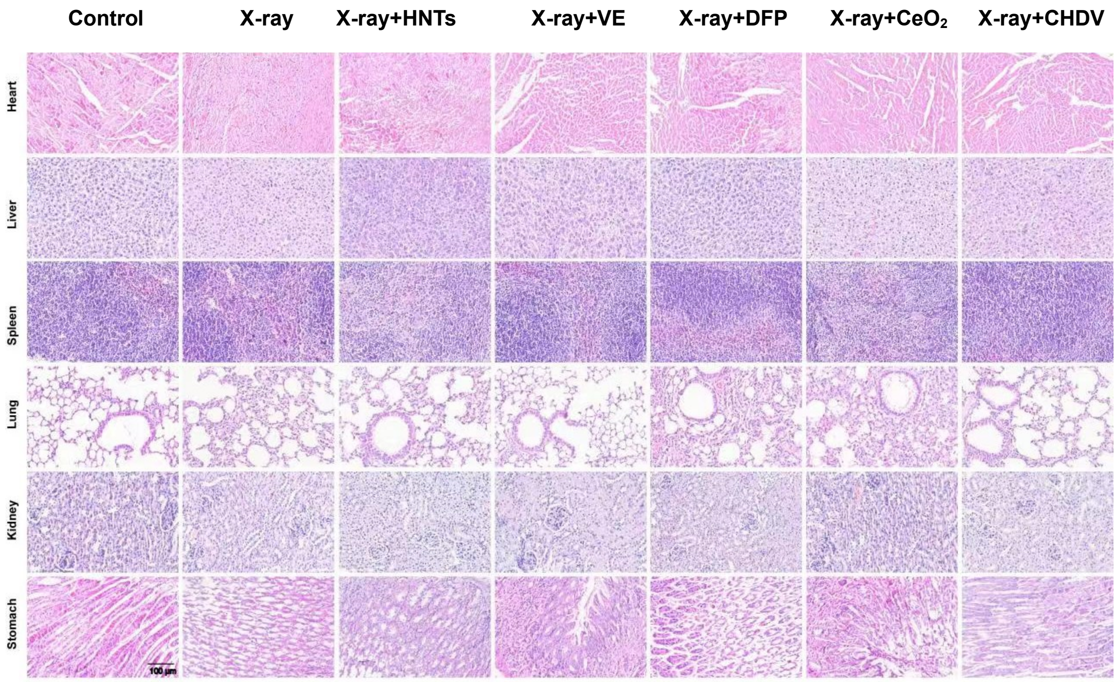

Supplementary Fig. 27. Representative H&E staining of main organ sections in each group after radiation colitis model treatment (*n* = 6 biologically independent animals). Scale bars: 100 μm. Experiment was repeated three times independently with similar results.

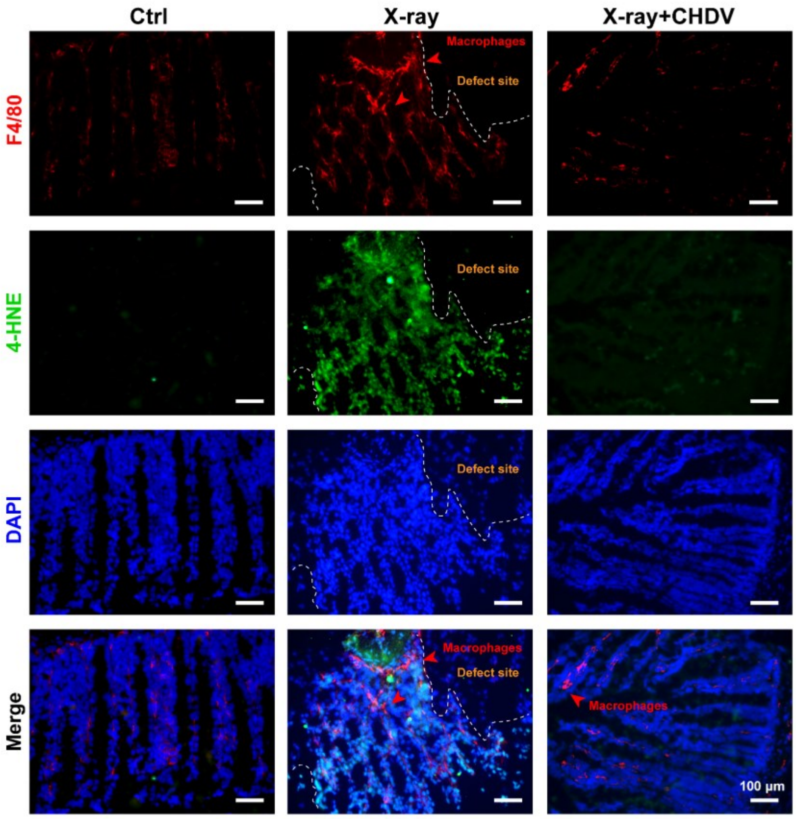

Supplementary Fig. 28. Immunofluorescence images of macrophages (F4/80, red), lipid peroxidation (4-HNE, green) and DAPI (nuclei, blue) in colonic sections after irradiation with or without therapeutic agent treatment (*n* = 3 biologically independent animals). Scale bars: 100 μm. Experiment was repeated three times independently with similar results.

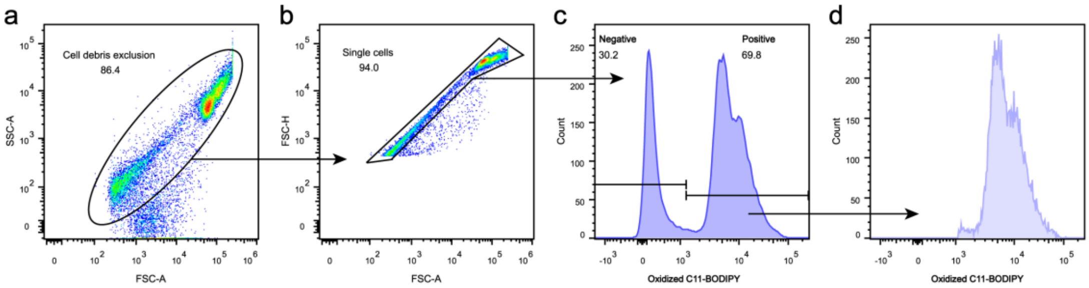

Supplementary Fig. 29. Gating strategy for analysis of lipid peroxidation in the IEC-6 cells, which was presented in Figure 2**j**, 2**k**, 5**c**, and Supplementary Fig. 3**a**, 3**b**. **a**) Exclusion of cell debris. **b**) Selective analysis of single cells (10,000 single cells were collected for further analysis). **c**) Analysis of oxidized C11-BODIPY. **d**) Identification of the lipid peroxidation-positive population. Gate **c** was used for mean fluorescent intensity statistics, and Gate **d** was used for positive intensity comparison between different groups.

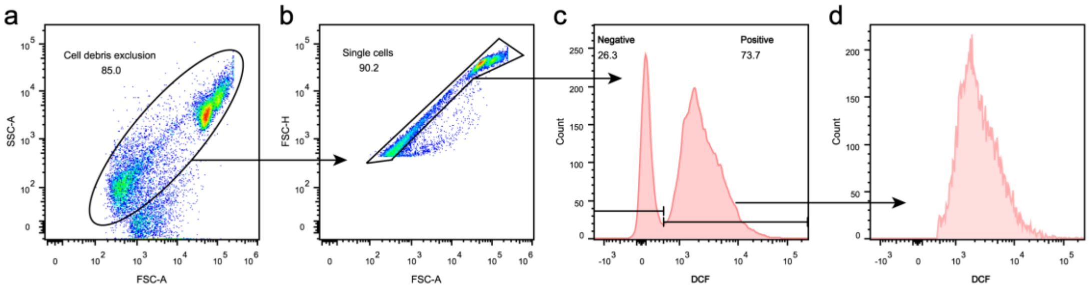

Supplementary Fig. 30. Gating strategy for analysis of ROS in the IEC-6 cells, which was presented in Supplementary Fig. 2**b**, 3**d**, 3**e**, and Supplementary Fig. 19**b**. **a**) Exclusion of cell debris. **b**) Selective analysis of single cells (10,000 cells were collected for further analysis). **c**) DCF analysis. **d**) Identification of the ROS-positive population. Gate **c** was used for mean fluorescent intensity statistics, and Gate **d** was used for positive intensity comparison between different groups.

Supplementary Tables

Supplementary Table 1. Binding energy of the Ce 3d states of the ceria in XPS

**Ce^{4+} (eV)**		**Ce^{3+} (eV)**	
V	882.7	V_0	879.0
V^{**}	888.8	V^{*}	885.6
V^{***}	898.8	U_0	897.9
U	900.8	U^{*}	903.3
U^{**}	907.6		
U^{***}	917.0		

Supplementary Table 2. Sequences for q-PCR primers

Gene	Base sequence (5'-3')
Mice *Ptgs2* forward	5′-AGCCCATTGAACCTGGACTG-3′
Mice *Ptgs2* reverse	5′-ACCCAATCAGCGTTTCTCGT-3′
Mice *Acsl4* forward	5′-CCTCCCCAGTACTGCAACAG-3′
Mice *Acsl4* reverse	5′-GGCTGAGAATTCGTGCATGG-3′
Mice *Fth1* forward	5′-TAAAGAACTGGGTGACCACGTGAC-3′
Mice *Fth1* reverse	5′-AAGTCAGCTTAGCTCTCATCACCG-3′
Mice *Ftl* forward	5′-TGGCCATGGAGAAGAACCTGAATC-3′
Mice *Ftl* reverse	5′-GGCTTTCCAGGAAGTCACAGAGAT-3′
Mice *Gpx4* forward	5′-CCTCCCCAGTACTGCAACAG-3′
Mice *Gpx4* reverse	5′-GGCTGAGAATTCGTGCATGG-3′
Mice *Tnf-α* forward	5′-CATCTTCTCAAAATTCGAGTGACAA-3′
Mice *Tnf-α* reverse	5′-TGGGAGTAGACAAGGTACAACCC-3′
Mice *Il6* forward	5′-AGACTTCCATCCAGTTGCCTTCTTG-3′
Mice *Il6* reverse	5′-TAAGCCTCCGACTTGTGAAG-3′
Mice *Cxcl1* forward	5′-CACAGGGGCGCCTATCGCCAA-3′
Mice *Cxcl1* reverse	5′-CAAGGCAAGCCTCGCGACCAT-3′
Mice *Gapdh* forward	5′-TCAAGAAGGTGGTGAAGCAG-3′
Mice *Gapdh* reverse	5′-GTTGAAGTCGCAGGAGACAA-3′

Supplementary Table 3. Clinical features of 5 patients with radiation colitis

Number	Primary carcinoma	Fractionated radiotherapy, Times	Clinical diagnosis	Symptom	Duration between the first symptom to the last radiotherapy, Months	Duration between the last radiotherapy and radiation colitis surgery, Months
**1** (Supplementary Fig. 1)	Endometrial cancer	28	Radiation colitis	Hematochezia	2	5
**2** (Supplementary Fig. 1)	Endometrial cancer	6	Radiation colitis	Increased stool frequency, hematochezia, anal pain	4	7
**3** (Supplementary Fig. 1)	Cervical cancer	25	Radiation colitis	Increased defecation frequency, hematochezia,	0, 6	13
**4** (Supplementary Fig. 1)	Cervical cancer (IIA)	25	Radiation colitis	Increased defecation frequency, dark red bloody stools	1	4
**5** (Figure 2b)	Cervical cancer (IB)	25	Radiation colitis	Abdominal pain, anal pain, increased frequency of defecation, vaginal defecation	8	9
