# Supplementary material for: A ferroptosis-targeting ceria anchored halloysite as orally drug delivery system for radiation colitis therapy
Source: Nat Commun. 2023 Aug 22;14:5083. doi: 10.1038/s41467-023-40794-w (PMC10444825; doi:10.1038/s41467-023-40794-w)
Supplement: Supplementary file 3 — Reporting Summary [file 41467_2023_40794_MOESM3_ESM.pdf]

nature portfolio

Corresponding author(s): Mingxian Liu, NCOMMS-22-47665

Last updated by author(s): Aug 10, 2023

Reporting Summary

Nature Portfolio wishes to improve the reproducibility of the work that we publish. This form provides structure for consistency and transparency in reporting. For further information on Nature Portfolio policies, see our Editorial Policies and the Editorial Policy Checklist.

Statistics

For all statistical analyses, confirm that the following items are present in the figure legend, table legend, main text, or Methods section.

n/a	Confirmed	
☐	☒	The exact sample size (n) for each experimental group/condition, given as a discrete number and unit of measurement
☐	☒	A statement on whether measurements were taken from distinct samples or whether the same sample was measured repeatedly
☐	☒	The statistical test(s) used AND whether they are one- or two-sided<br>*Only common tests should be described solely by name; describe more complex techniques in the Methods section.*
☒	☐	A description of all covariates tested
☒	☐	A description of any assumptions or corrections, such as tests of normality and adjustment for multiple comparisons
☐	☒	A full description of the statistical parameters including central tendency (e.g. means) or other basic estimates (e.g. regression coefficient) AND variation (e.g. standard deviation) or associated estimates of uncertainty (e.g. confidence intervals)
☐	☒	For null hypothesis testing, the test statistic (e.g. F, t, r) with confidence intervals, effect sizes, degrees of freedom and P value noted<br>*Give P values as exact values whenever suitable.*
☒	☐	For Bayesian analysis, information on the choice of priors and Markov chain Monte Carlo settings
☒	☐	For hierarchical and complex designs, identification of the appropriate level for tests and full reporting of outcomes
☒	☐	Estimates of effect sizes (e.g. Cohen's d, Pearson's r), indicating how they were calculated

Our web collection on statistics for biologists contains articles on many of the points above.

Software and code

Policy information about availability of computer code

Data collection	Flow Cytometry: FACSDiva (6.1). Fluorescence microscope: ZEN lite (2.0.15), Microplate reader: Gen5 (2.0).
Data analysis	GraphPad Prism (8.0), Origin (2021), FlowJo (V10.0), ImageJ (1.8.0), ViewPoint (2.0.2).

For manuscripts utilizing custom algorithms or software that are central to the research but not yet described in published literature, software must be made available to editors and reviewers. We strongly encourage code deposition in a community repository (e.g. GitHub). See the Nature Portfolio guidelines for submitting code & software for further information.

Data

Policy information about availability of data

All manuscripts must include a data availability statement. This statement should provide the following information, where applicable:

- Accession codes, unique identifiers, or web links for publicly available datasets
- A description of any restrictions on data availability
- For clinical datasets or third party data, please ensure that the statement adheres to our policy

The data that support the findings of this study are available within the main text and its Supplementary Information file. Source data is provided as Source Data file. Source data are provided with this paper.

Human research participants

Policy information about studies involving human research participants and Sex and Gender in Research.

Reporting on sex and gender	The findings of this study do not apply to only one sex or gender. Gender and sex were not considered in the study design. This is because there are limited reports indicating any association between sex or gender and the occurrence and development of radiation colitis. The radiation-induced damage to the intestines is a general phenomenon and is not influenced by sex or gender differences.
Population characteristics	Patients with a clinical diagnosis of radiation colitis following pelvic radiotherapy.
Recruitment	Patients requiring treatment for radiation-induced colitis were recruited, and the research team contacted them to obtain informed consent. These patients typically presented symptoms such as rectal bleeding and increased bowel frequency. The selection of participants was not based on the severity of their condition in order to minimize bias.
Ethics oversight	Human Medical Ethics Committee of the Sixth Affiliated Hospital of Sun Yat-sen University.

Note that full information on the approval of the study protocol must also be provided in the manuscript.

Field-specific reporting

Please select the one below that is the best fit for your research. If you are not sure, read the appropriate sections before making your selection.

☒ Life sciences ☐ Behavioural & social sciences ☐ Ecological, evolutionary & environmental sciences

For a reference copy of the document with all sections, see nature.com/documents/nr-reporting-summary-flat.pdf

Life sciences study design

All studies must disclose on these points even when the disclosure is negative.

Sample size	The calculation was based on the primary outcome measure and the anticipated effect size, variability, and statistical tests to be used. In vitro cell experiments, due to the stable nature of the culture system and the relatively low cost of repetition, were performed with 3 or 4 independent biological replicates (refer to Cell Death & Differentiation, 2021, 28(6): 1971-1989). Animal experiments, due to the presence of individual variability, high cost of repetition, and the possibility of unpredictable unexpected deaths leading to data loss, were conducted with a minimum of 5 independent biological replicates (refer to Nature chemical biology, 2021, 17(4): 465-476). In this study, we prepared 10 mice per group for testing the treatment efficacy, and an additional 10 mice per group for survival analysis.
Data exclusions	No exclusions of data were made that would significantly impact the results or conclusions, reported in Experimental design of section Methods.
Replication	At least three biological replicates were performed for each finding to ensure reproducibility. All attempts at replication were successful.
Randomization	Sample allocation to each experimental group was randomized.
Blinding	The researchers responsible for administering gastric administration were aware of the animal grouping as different drug treatments were applied based on the groups. However, the researchers collecting and analyzing the data were unaware of the group assignments and followed a blind procedure.

Reporting for specific materials, systems and methods

We require information from authors about some types of materials, experimental systems and methods used in many studies. Here, indicate whether each material, system or method listed is relevant to your study. If you are not sure if a list item applies to your research, read the appropriate section before selecting a response.

Materials & experimental systems

n/a	Involved in the study
☐	☒ Antibodies
☐	☒ Eukaryotic cell lines
☒	☐ Palaeontology and archaeology
☐	☒ Animals and other organisms
☒	☐ Clinical data
☒	☐ Dual use research of concern

Methods

n/a	Involved in the study
☒	☐ ChIP-seq
☐	☒ Flow cytometry
☒	☐ MRI-based neuroimaging

Antibodies

Antibodies used

Phospho-Histone H2AX antibody (Cell Signaling Technology, Cat. #2577S, 20E3),
Anti-Rabbit IgG (H+L) F(ab')2 Fragment (Alexa Fluor® 488 Conjugate)(Cell Signaling Technology, Cat. #4412S),
Anti-4 Hydroxynonenal antibody (Abcam, ab46545),
Anti-Ferritin Heavy Chain antibody (Abcam, ab65080),
Anti-beta Actin antibody (Abcam, ab6276),
Ferritin light chain Polyclonal antibody (Proteintech, 10727-1-AP),
ACSL4 Rabbit mAb (Zenbio, R24265),
Anti-Glutathione Peroxidase 4 antibody (Abcam, ab125066),
Cleaved-Caspase 3 p17 Rabbit pAb (Zenbio, R23726),
Anti-F4/80 antibody (Abcam, ab16911),
HRP AffiniPure Goat Anti-Rabbit IgG (H+L) (Fude biotech, FDR007)
HRP AffiniPure Goat Anti-Mouse IgG (H+L) (Fude biotech, FD0142)
Donkey anti-Rat IgG (H+L) Secondary Antibody, TRITC (Invitrogen, A18744)
Goat anti-Rabbit IgG (H+L) Cross-Adsorbed Secondary Antibody, Alexa Fluor™ 488 (Invitrogen, A11008)
Goat anti-Rabbit IgG (H+L) Cross-Adsorbed Secondary Antibody, Alexa Fluor™ 555 (Invitrogen, A21428)

Validation

Phospho-Histone H2AX antibody (Cell Signaling Technology, Cat. #2577S): rat IF (Cell Death Discovery, 2021, 7(1): 356).
Anti-4 Hydroxynonenal antibody (Abcam, ab46545): mice WB (Journal of neuroinflammation, 2017, 14(1): 1-17), mice IF (Cell reports, 2020, 30(10): 3411-3423. e7), mice IHC (Biomedicine & Pharmacotherapy, 2020, 128: 110306). Human IHC (Redox biology, 2021, 43: 102006).
Anti-Ferritin Heavy Chain antibody (Abcam, ab65080): mice WB (Science Advances, 2021, 7(51): eabl5862).
Anti-beta Actin antibody (Abcam, ab6276): WB (Nature Communications, 2022, 13(1): 6744) , mice WB (ImmunoHorizons, 2021, 5(10): 818-829), (Aging and Disease, 2022, 13(6): 1875).
Ferritin light chain Polyclonal antibody (Proteintech, 10727-1-AP): mice WB (ACS nano, 2023, 17(3): 2440-2449), rat WB (Translational Research, 2021, 229: 53-68).
ACSL4 Rabbit mAb (Zenbio, R24265): mice WB (Biomaterials, 2021, 277: 121103), rat WB (Sleep and Breathing, 2023: 1-8). IHC (Verification provided by the manufacturer: http://www.zen-bio.cn/prod_view.aspx?IsActiveTarget=True&TypeId=171&Id=557595&FId=t3:171:3).
Anti-Glutathione Peroxidase 4 antibody (Abcam, ab125066): mice WB (Cell Death & Disease, 2022, 13(11): 1006).
Cleaved-Caspase 3 p17 Rabbit pAb (Zenbio, 341034): IF (Brain Research Bulletin, 2022, 189: 139-150), mice WB (Chemical Engineering Journal, 2021, 426: 130827).
Anti-F4/80 antibody (Abcam, ab16911): mice IF (Proceedings of the National Academy of Sciences, 2013, 110(21): 8674-8679).

Eukaryotic cell lines

Policy information about cell lines and Sex and Gender in Research

Cell line source(s)	Rat intestinal epithelial cell line IEC-6 was obtained from the American Type Culture Collection.
Authentication	The IEC-6 cell line authentication has performed using STR (Short Tandem Repeat) analysis to confirm its identity and uniqueness.
Mycoplasma contamination	The IEC-6 cell line has tested and confirmed to be negative for Mycoplasma contamination.
Commonly misidentified lines (See ICLAC register)	IEC-6 cells are not listed as commonly misidentified lines in the ICLAC register.

Animals and other research organisms

Policy information about studies involving animals; ARRIVE guidelines recommended for reporting animal research, and Sex and Gender in Research

Laboratory animals	Female C57BL/6J mice, 8 weeks old. Mice were housed in a SPF-grade animal facility with a constant temperature of 24±1°C, humidity of 51±5%, and a 12-hour light/12-hour dark cycle. The mice were housed in groups of five per cage and had ad libitum access to food and water, ensuring animal welfare.
Wild animals	This study did not involve wild animals.

Reporting on sex	The results do not apply to only one sex. The animal models involved in this study and the results were not affected by sex.
Field-collected samples	This study did not involve samples collected from the field.
Ethics oversight	All animal care and experimental procedures were followed in accordance with guidelines approved by the Ethics Committee of Jinan University Laboratory Animal Center (approval number: 20210528-11). In this study, the body weight of the mice did not exceed a decrease of 20% from normal values, meeting the requirements of the Ethics Committee. The mice were anesthetized using pentobarbital sodium and euthanized by overdose anesthesia.

Note that full information on the approval of the study protocol must also be provided in the manuscript.

Flow Cytometry

Plots

Confirm that:

☒ The axis labels state the marker and fluorochrome used (e.g. CD4-FITC).

☒ The axis scales are clearly visible. Include numbers along axes only for bottom left plot of group (a 'group' is an analysis of identical markers).

☒ All plots are contour plots with outliers or pseudocolor plots.

☒ A numerical value for number of cells or percentage (with statistics) is provided.

Methodology

Sample preparation	The intestinal epithelial cell line IEC-6 was collected at specific time points after treatment with drugs, irradiation, etc. For lipid peroxidation analysis, cells were digested with trypsin and stained with C11-BODIPY for 30 minutes. After one wash with serum-free medium, the corresponding fluorescence was detected by a flow cytometry. For reactive oxygen species analysis, the dye replaced with DCFH-DA, following the same procedure as mentioned above.
Instrument	BD FACSCanto ll, no special making.
Software	Data collection were performed using FACSDiva™ software, and analyze were performed by FlowJo. V10.
Cell population abundance	Cell lines with a homogeneous population were employed in this study. Therefore, 10,000 cells per tube were collected for the analysis of oxidized C11-BODIPY (lipid peroxidation) and DCF (intracellular reactive oxygen species).
Gating strategy	A instrument threshold (1000) was set to automatically exclude cell debris, followed by manual gating in the FSC/SSC panel to remove any remaining cell debris. Single cell populations are displayed using both FSC-H and FSC-A parameters (to avoid analyzing agglomerates).

☒ Tick this box to confirm that a figure exemplifying the gating strategy is provided in the Supplementary Information.
